# Menopause Symptom Experience and Perceived Well-Being Among HIV Positive and Negative Older Women in Ibadan, Nigeria

**DOI:** 10.1093/geroni/igaa057.732

**Published:** 2020-12-16

**Authors:** Olubukola Omobowale, Olubukola Adesina

**Affiliations:** 1 University of Ibadan, Nigeria, Ibadan, Oyo, Nigeria; 2 University of Ibadan, Ibadan, Oyo, Nigeria

## Abstract

Globally, people are living longer with the Human Immunodeficiency Virus (HIV) and older individuals are becoming infected. Menopause symptoms affect women’s health and are associated with perceived declines in wellbeing. This study assessed and compared the menopause symptom experience and perceived wellbeing among HIV positive and negative older women in Ibadan Nigeria Focus group discussions were conducted among menopausal women attending the ARV and GOP clinics at the University College Hospital Ibadan. Opinions of discussants on knowledge and experience of menopausal symptoms, perceptions about the menopause and perceived health status were explored. A total of 90 HIV positive and 92 HIV negative women aged between 40 to 60 years were sampled. Knowledge of the cause of menopause was poor, with more HIV positive women opining that sexual promiscuity causes menopause. The majority of the discussants had adequate knowledge of menopausal symptoms with most of them reporting vasomotor and musculoskeletal symptoms. In both groups, perceptions about the menopause were generally positive as most of them opined that the menopause means freedom from sexual activity and child birth. More HIV negative women perceived themselves to be in good health compared to HIV positive women. Menopause induces many of the same metabolic changes that are being observed with HIV infection, and this may affect the health and quality of life of aging women with HIV infection. There’s a need for health education and health promoting interventions that will help these women in coping with the double burden of HIV infection and menopause.

